# Occurrence and Human Health Risk of Dichlorodiphenyltrichloroethane (DDT) and Hexachlorocyclohexane (HCH) Pesticide Residues in Commonly Consumed Vegetables in Southwestern Nigeria

**DOI:** 10.5696/2156-9614-9.23.190909

**Published:** 2019-08-06

**Authors:** Adeoluwa Oluwaseyi Adeleye, Mosudi Babatunde Sosan, John Adekunle Oyedele Oyekunle

**Affiliations:** 1 Department of Crop Production and Protection, Obafemi Awolowo University, Ile-Ife, Nigeria; 2 Department of Chemistry, Obafemi Awolowo University, Ile-Ife, Nigeria

**Keywords:** DDT, HCH, pesticide residues, fluted pumpkin, amaranths, health risk, safety

## Abstract

**Background.:**

Fluted pumpkin (Telfairia occidentalis) and amaranth (Amaranthus spp.) are common leafy vegetables produced and consumed in southwestern Nigeria. These vegetables attract insect pests which are controlled by pesticides that may have adverse impacts on human health.

**Objectives.:**

To determine the levels of dichlorodiphenyltrichloroethane (DDT) and hexachlorocyclohexane (HCH) pesticide residues in the two vegetables and evaluate the potential health risks associated with their consumption.

**Methods.:**

The pesticide residue levels were quantitatively and qualitatively determined using a gas chromatograph coupled with electron capture detector. Health risk assessment were performed using estimated average daily intake and hazard indices for two weight categories: children (16.7 kg) and adults (60 kg).

**Results.:**

The results showed that *delta*-HCH, DDT and methoxychlor were predominantly detected in the two vegetables from both farms and markets. In amaranth, the mean concentration of methoxychlor, DDT and *delta*-HCH were 4.590 ± 2.774 mg/kg (dry weight (dw)), 0.757 ± 0.457 mg/kg (dw) and 0.577 ± 0.390 mg/kg (dw), respectively, while fluted pumpkin levels were 6.223 ± 2.489 mg/kg dw (methoxychlor), 0.504 ± 0.056 mg/kg dw (*delta*-HCH) and 0.486 ± 0.123 mg/kg dw (DDT). The levels of HCH and DDT residues were generally above the United Kingdom/European Commission maximum residue limit. The analysis of health risk estimates for non-carcinogenic risk revealed that for both vegetables, the hazard quotient for *p, p′* DDT and methoxychlor was >1 for both children and adults. The health risk estimates for carcinogenic risk revealed that hazard indices values were >1 for children for both vegetables and *alpha*-HCH had a hazard index >1 for adults for amaranth. This means the residue exceeds acceptable standard and present potential risk to consumers of these vegetables.

**Conclusions.:**

The results obtained from the present study indicate that consumption of amaranth contaminated with *alpha*-HCH could pose a carcinogenic risk for adult consumers. The consumption of the two vegetables could pose both non-carcinogenic and carcinogenic health risks to children and adults. Therefore, there is need for strict enforcement of regulations on pesticide usage to minimize human health risks.

**Competing Interests.:**

The authors declare no competing financial interests.

## Introduction

Fluted pumpkin (Telfairia occidentalis Hook F.) and amaranth (Amaranthus spp.) are leafy vegetables which are widely cultivated and commonly consumed in southwestern Nigeria. Nigeria is the fourth highest producer of vegetables in the world and highest producer in Africa.^1^ Vegetables are good sources of fiber, minerals, vitamins and antioxidants.^2^,^3^ Apart from their nutritional value, they can be a source of toxic substances such as pesticide residues which are caused by indiscriminate use, misuse or overuse of pesticides to control the high incidence of infestation of pests in vegetables.^4^

Dichlorodiphenyltrichloroethane (DDT) and hexachlorocyclohexane (HCH) are organochlorine pesticides (OCPs) and also regarded as persistent organic pollutants.^5^ They have remarkably toxic properties and acute poisoning tendency with anti-androgenic activities.^6^ Human exposure to these pesticides occurs mainly from residues in food and the level of exposure depends on both the quantity of food consumed and residue levels, with impacts ranging from acute to chronic health hazards. Studies have shown that exposure to HCHs and DDT over a short period can produce dizziness, headache, nausea, vomiting, tremors, convulsions, and muscle weakness, and long-term exposure has been reported to result in health effects such as endocrine disruption, behavioral changes, blood disorders, Parkinson's disease, reproductive defects, neurological problems, immunologic, teratogenic disorders, and cancer in organisms and humans.^7^–^10^

Pesticide residues such as HCHs and DDT have been reported in amaranth, cabbage, lettuce, pumpkin and spinach in China; and in cabbage, lady's finger, tomato and brinjal in India.^11^,^12^ In Nigeria, the presence of HCH and DDT residues have also been reported in vegetables such as amaranth by Adeyeye and Osinbajo, spinach, lettuce, and cabbage by Akan *et al*. and fluted pumpkin by Ibrahim *et al*.^13^–^15^ In other food products in Nigeria, HCH and DDT have been reported in cowpea and yam chips by Sosan *et al.*, and cocoa beans by Oyekunle *et al.*^16^,^17^ Risk assessment of pesticide residues in food products are routinely carried out in developed countries to ascertain contamination resulting from pesticide application and suitability for consumption. However, in Nigeria, only two studies were identified. A study by Oyeyiola *et al*. assessed the potential health risk of contaminated vegetables in Lagos such as cabbage, carrot, pepper, tomatoes and lettuce, and a study by Sosan and Oyekunle assessed the potential health risk from kolanut consumption.^18^,^19^ There is a dearth of information on the potential health risk associated with commonly consumed vegetables such as amaranth (Amaranthus spp.) and fluted pumpkin (T. occidentalis). In order to guarantee that vegetables produced and marketed are safe for consumption, the present study was undertaken to evaluate the levels of pesticide residues and associated health risks in two leafy vegetables collected from selected farms and markets in Osun and Ekiti States in southwestern Nigeria.

**Abbreviations**
*DCM*: Dichloromethane
*DDD*: Dichlorodiphenyldichloroethane
*DDE*: Dichlorodiphenyldichloroethylene
*DDT*: Dichlorodiphenyltrichloroethane
*dw*: Dry weight
*EADI*: Estimated average daily intake
*HCH*: Hexachlorocyclohexane
*HQ*: Hazard quotient
*MRLs*: Maximum residue limits
*OCPs*: Organochlorine pesticides

## Methods

**Figure 1:**
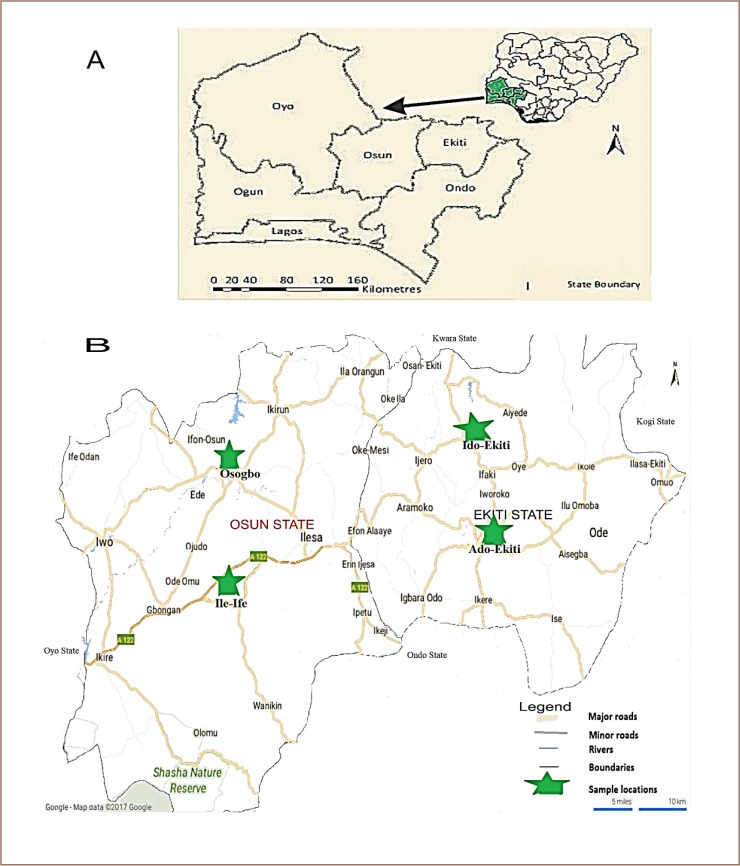
Study area showing sampling locations in southwestern Nigeria

The leafy vegetable samples (amaranth and fluted pumpkin) used for the present study were obtained from selected farms and open markets in Ado-Ekiti (7° 37′ N and 5° 13′ E) and Ido-Ekiti (7° 50′ N and 5° 10′ E), Ile-Ife (70° 50′ N and 4° 69′ E) and Osogbo (7° 46′ N and 4° 34′ E) in southwestern Nigeria *(Figure 1).* The sampling sites in selected locations from Ekiti State were Ogbese, Erinfun, Odo-Ado, Akuro Alapo, Igboata farms, and Awedele, Oja-oba, Oja Bisi, Olojudo and Ido daily markets. In Osun State, selected locations were Ede road, Oju-ako, Oke-osun and Osun Agricultural farms, and Oluode, Alekuwodo, Obafemi Awolowo University Central and Ile-Ife Central markets. The two states were selected based on their agrarian nature and the locations or towns were selected based on populations of vegetable farmers, their production levels and size of markets. Each of the vegetable types were purchased from three randomly selected market stalls and farms within sampling sites (November 2017–January 2018). Vegetable samples collected from each location were wrapped in aluminum foil to avoid cross contamination. The foil was wiped clean with cotton wool soaked in acetone. A total of 96 samples (48 for each of the vegetables) were later bulked into 32 composite samples and labelled before being transferred to the laboratory.

### Sample preparation

Each vegetable sample was rinsed with distilled water, allow to drain properly and chopped with a clean sharp knife on a chopping board and oven dried at 45°C for three days until a constant weight was attained. To obtain a homogenous representative sample, each composite sample was macerated and pulverized to a homogenous powdered form using a Nakai blender (Japan). The knife, chopping board and blender were washed thoroughly with water and rinsed with acetone to avoid cross contamination. Each sample was then placed in Ziploc bags, well labelled and stored in a cool place prior to further analysis.

### Extraction of pesticide residues from samples

For each sample, 20 g of homogenized vegetable was weighed into a pre-extracted Whatman thimble. Following the method of Oyekunle *et al.*; the sample was Soxhlet extracted for 4 hours using dichloromethane (DCM) as the extraction solvent.^20^ The extract was then concentrated by distilling-off the solvent (DCM) on a rotary evaporator at about 41°C. The reduced extract was then preserved for clean-up.

### Clean-up

A clean-up experiment was carried out to separate analytes from interfering compounds of a different polarity or removal of impurity (chlorophyll) from extracts, a column of about 15 cm (length) × 1 cm (internal diameter) was packed first with glass wool and then 5 g activated silica gel prepared in a slurry form in DCM. About 5 g of anhydrous sodium sulfate was placed on top of the column to absorb any water in the sample or the solvent. The pre-elution was done with 15 mL of DCM without exposing the sodium sulfate layer to air so as to prevent the cracking of packed silica gel adsorbent. The reduced extracts were run through the column and allowed to sink below the sodium sulfate layer. Elution was done with 3 × 10 mL portions of DCM.

The eluents were collected and the accompanying solvent was then evaporated to dryness under a stream of pure nitrogen (99.99%).

### Instrumental analysis

The instrumental analysis was carried out at the Nigeria Institute of Oceanography and Marine Research Laboratory, Lagos, Nigeria. Detection and determination of the pesticide residues were performed by reconstituting the dried sample eluents with 2 mL n-hexane before injecting 1 μL of the cleaned-up eluents into injection port of an Agilent 7890A gas chromatograph system equipped with an electron capture detector. The separation was performed on a fused silica capillary column (DB-17, 30 m × 0.250 mm internal diameter and film thickness of 0.25 μm). The temperatures of the injector and detector were 250°C and 290°C, respectively. Oven temperature started at 150°C and increased to 280°C at 6°C per minute. The injection was through a splitless injector, using helium as a carrier gas at a flow rate of 2 mL/min and nitrogen as a make-up gas. The run time was 21.67 minutes. Quantification of the OCPs was based on external calibration curves prepared from the standard solutions of alpha (α)-HCH, beta (β)-HCH), delta (δ)-HCH, gamma (γ)-HCH, dichlorodiphenyldichloroethane (DDD), dichlorodiphenyldichloroethylene DDE), DDT, and methoxychlor.

### Quality assurance and control

All analytical procedures were monitored using strict quality assurance and control measures. Materials used for preparation of samples were well washed and rinsed with acetone before re-use. Chemicals used in sample preparation and analyses were of analytical grade. Blank determination, percentage recovery determination, response factor, and limit of detection determination were also carried out for quality control and assurance.

### Blank determinations

The background value of OCPs in the analytical grade DCM and eluent of the pre-extracted thimble was determined by injecting 1 μL of the cleaned-up eluents and solvent into the gas chromatograph coupled with electron capture detector. No organochlorine pesticide residue was detected on blank samples.

### Recovery determination

Four samples of homogenized vegetable, each weighing 20 g, were chosen. For each vegetable, one sample was spiked with 10 mg kg^−1^ standard mixture consisting of some of the available organochlorine pesticides of interest. The mixture was thoroughly mixed together to ensure maximum homogenization. The other two vegetable samples were left un-spiked. The samples (amaranth and fluted pumpkin) were extracted and cleaned up following procedures of Oyekunle *et al*. and followed by gas chromatograph coupled with electron capture detector analysis.^20^ The recoveries were determined by comparing the peak areas of the OCPs after spiking with those un-spiked. Percentage recoveries were evaluated based on Equation 1.

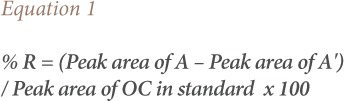
where, %R = percentage recoveries, A = OCP in spiked sample and A′= OCP in un-spiked sample.

The percent recovery obtained was within the 70–120% range for acceptable recovery values stipulated by the European Union's guidelines for evaluating the accuracy and precision of a method.^21^ This suggests that the procedure outlined for this study can be considered reliable and efficient.

### Response factor determination

The response factor of the standard OCPs was obtained by the method of Sosan and Oyekunle.^19^ This was determined by analyzing 1.0 μL of 1000 ppm stock solution of the standard mixture containing the internal standard. The internal standard used for this work was hexachlorobenzene. The response factor for a sample peak is defined by Equation 2:

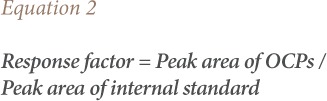



The limit of detection of each of the OCPs was evaluated using the relationship: limit of detection = 3.3 S/b, where S is the residual standard deviation of the calibration function and b is the slope of the calibration curve.^22^

### Statistical analysis

The results obtained from the chromatographic analysis were summarized using descriptive statistics (mean, range, standard error and percentages) and charts on a log (x+1) scale and normal scale. The Wilcoxon (Mann-Whitney U test) test was conducted to examine differences between market and farm samples of amaranths and fluted pumpkin using Statistical Analysis System (SAS) 9.0 version.

### Health risk estimation

Data on pesticide residues levels were compared with maximum residue limits (MRLs) recommended by the United Kingdom/European Commission (UK/EC) for leafy vegetables.^23^ The health risk estimates for each of the organochlorine pesticide residues detected in the vegetables was computed using two basic standard indices: estimated average daily intake (EADI) and health risk index.

The EADIs of a pesticide residues and the food consumption assumption were used to determine long term health risks to consumers. The EADI was obtained by multiplying the mean residual pesticide concentration (mg kg^−1^) in the food of interest and the food consumption rate (kg d^−1^) and dividing by body weight.^19^

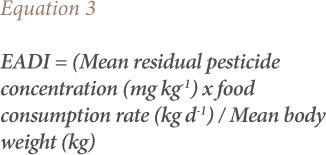



The consumption rate for vegetables for adults was considered to be 89.3 g/person/day and 140 g/person/day for children.^18^,^24^,^25^ The potential non-carcinogenic health risk was assessed by calculating the hazard quotient.^18^,^19^

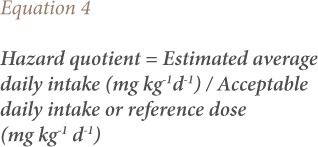



The average adult's body weight of 60 kg and 16.7 kg for children were considered as standard body weight.^18^,^26^ The reference dose was obtained from the United States Environmental Protection Agency Integrated Risk Information System.^27^ When the health risk index > 1, the food in question is considered to be a risk to consumers; when the index < 1, the food is considered acceptable for consumption.^18^,^19^

For the carcinogenic health risk, the hazard ratio was calculated using Equation 5.^28^,^29^

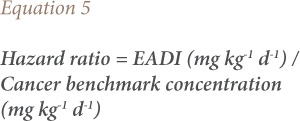
where,

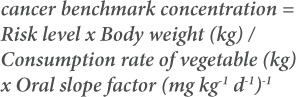



Risk is the maximum acceptable risk level (1 × 10^−6^). The cancer benchmark concentration for carcinogenic effect is derived by setting the risk to one in one million due to lifetime exposure. The oral slope factors for the pesticide were obtained from the United States Environmental Protection Agency (USEPA).^27^,^30^

## Results

Table 1 shows the percentage recovery, limit of detection and response factor values of various analytes or congeners in vegetable samples. Percentage recovery values for the pesticides were between (γ-HCH) 86.02% - (α-HCH) 107.5%. The response factor of the organochlorine pesticides ranged from 0.921 (DDE) to 1.66 (DDD) and the limit of detection of the organochlorine pesticides were in the range of 0.0069 to 0.0102 mg kg^−1^. The blank determination recorded no peak.

**Table 1:** Percentage Recovery, Limit of Detection, and Response Factor of Organochlorine Pesticides

**Organochlorine pesticides**	**Response factor**	**Amount (mg kg^−1^) of OCP used for spiking**	**Mean amount of OCP recovered**	**% Recovery**	**Limit of detection (mg kg^−1^)**
Hexachlorobenzene	-	-	9.360	93.60	-
α-HCH	1.318 ±0.020	10	10.75	107.50	0.0069
β-HCH	1.105 ±0.005	10	9.395	93.95	0.0086
γ-HCH	1.190 ±0.023	10	8.602	86.02	0.0072
δ-HCH	1.402 ±0.007	10	9.735	97.35	0.0102
*p*, *p′* DDD	1.660 ±0.105	10	9.850	98.50	0.0074
*p*, *p′* DDE	0.921 ±0.005	10	8.807	88.07	0.0102
*p*, *p′* DDT	0.974 ± 0.009	10	9.225	92.25	0.0097
Methoxychlor	1.240 ±0.003	10	9.619	96.19	0.0091

**Figure 2:**
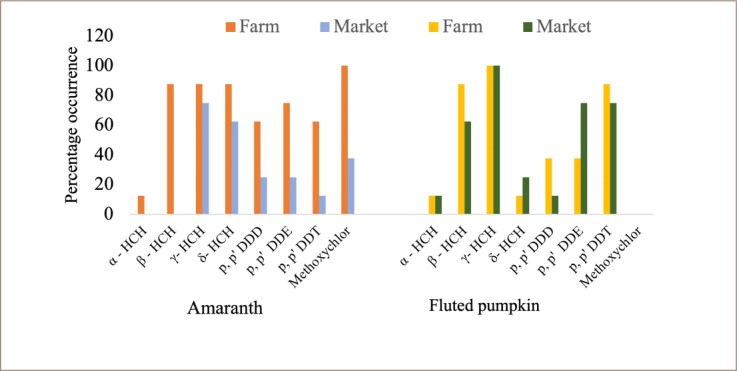
Percentage occurrence of DDTs and HCHs pesticide residues in amaranth and fluted pumpkin samples from selected farms and markets in southwestern Nigeria (representing data presented in Table 1 in Supplemental Material)

The levels of HCHs and DDTs in amaranth and fluted pumpkin from selected markets and farms in southwestern Nigeria are shown in Figure 2. All the DDTs and HCHs with their metabolites were detected in amaranths samples from farms. However, α-HCH and β-HCH were not detected in markets samples. In fluted pumpkin, all the compounds were detected in both farm and market samples with the exception of α-HCH. In amaranth, percentage occurrence ranged from 12.5 to 100% in samples collected from farms and ND -75% in market samples. In fluted pumpkin, percentage occurrence of the pesticide ranged from ND - 100% in both farm and market samples.

**Figure 3:**
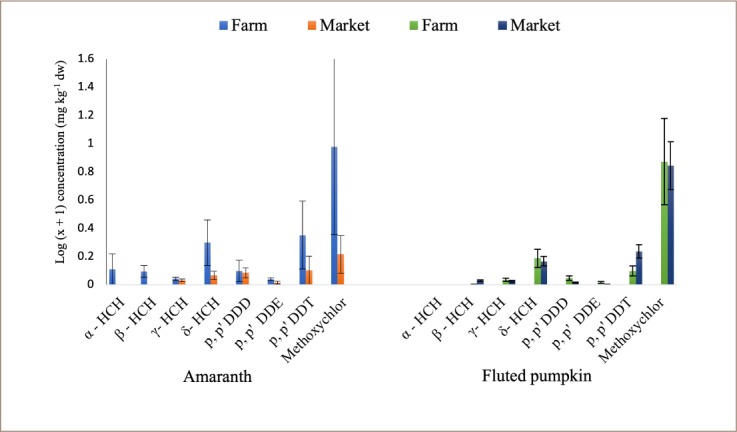
Mean concentration of DDT and HCH pesticide residues detected in amaranth and fluted pumpkin from selected farms and markets in southwestern Nigeria (representing data presented in Table 1 in Supplemental Material)

The mean concentration (log x+1 transformed) of DDTs and HCHs are presented in Figure 3. The highest mean concentrations of methoxychlor, DDT and δ-HCH of 8.536 ± 4.428 mg kg^−1^ (dry weight (dw)), 1.251 ± 0.737 mg kg^−1^ (dw), and 0.986 ± 0.448 mg kg^−1^ (dw), respectively, were detected in amaranth from farms and 0.643 ± 0.365 mg kg^−1^ (dw), 0.263 ± 0.263 mg kg^−1^ (dw) and 0.168 ± 0.068 mg kg^−1^ (dw), respectively, in market samples *(Supplemental Material, Table 1)*. Similar results were obtained for levels of residues detected in fluted pumpkin with methoxychlor, δ-HCH and DDT being the most prominent in both farm and market samples. The mean concentrations of methoxychlor, δ-HCH and DDT were 6.458 ± 1.821 mg kg^−1^ (dw), 0.542 ± 0.080 mg kg^−1^ (dw) and 0.254 ± 0.174 mg kg^−1^ (dw), respectively, for farm samples and 5.988 ± 2.533 mg kg^−1^ (dw), 0.467 ± 0.147 mg kg^−1^ (dw) and 0.718 ± 0.203 mg kg^−1^ (dw), respectively, for market samples *(Supplemental Material, Table 1)*. However, the levels of residues detected in amaranth from farms were higher than the levels in samples collected from markets, but not significantly different in fluted pumpkin collected from both markets and farms at p ≤ 0.05 (*Supplemental Material, Table 1*).

The ratio of α-HCH/γ-HCH detected in amaranth and fluted pumpkin was 1.6 and 0, respectively, while the ratio of (DDE+DDD)/DDT detected in amaranths and fluted pumpkin was 0.4 and 0.2, respectively *(Supplemental Material, Table 2)*.

**Figure 4:**
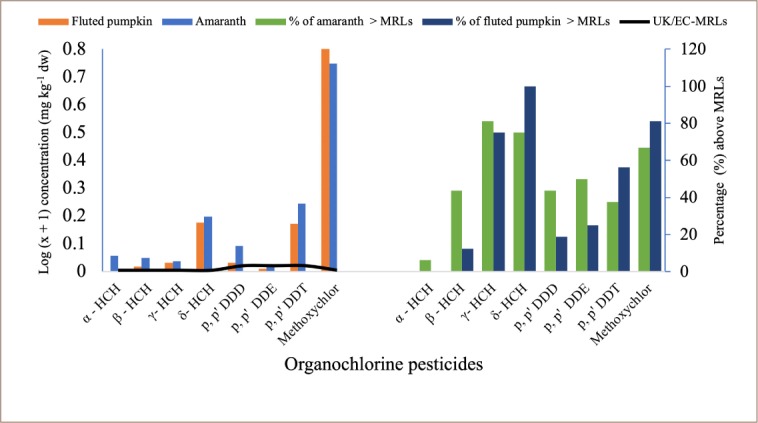
Maximum residue limits, mean concentration of organochlorine pesticide residues detected and percent of vegetable samples with residue levels above MRLs (representing data presented in Table 2 in Supplemental Material)

The mean concentration of pesticide residues detected in amaranth and fluted pumpkin in comparison with respective MRLs is presented in Figure 4. The mean concentration of pesticide residues detected in amaranth and fluted pumpkin were found to be above their respective recommended UK/EC maximum residue limit. In amaranth, the highest percentage of pesticide residue above MRLs was observed in γ-HCH (81.3%), δ-HCH (75%) and methoxychlor (66.8%), while in fluted pumpkin, δ-HCH (100%), methoxychlor (81.3%) and γ-HCH (75%) showed the highest percentage above MRLs. For α-HCH, only 6.3% of amaranths analyzed were above MRLs, and no α-HCH was detected in fluted pumpkin.

**Figure 5:**
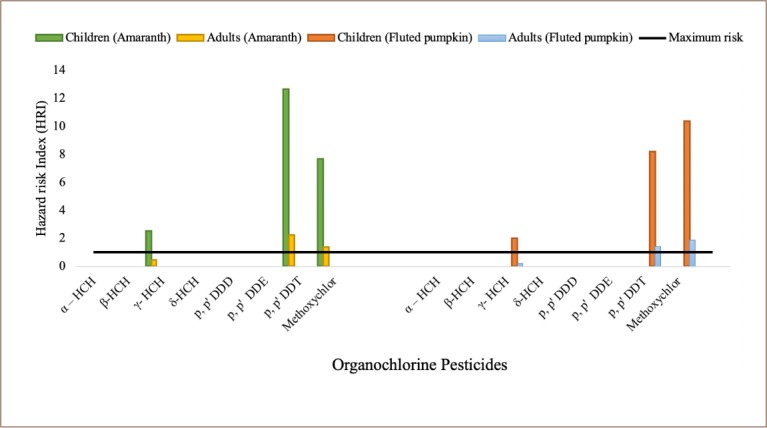
Potential non-carcinogenic health risk estimation of organochlorine residues in amaranth and fluted pumpkin from selected farms and markets in southwestern Nigeria (representing data presented in Table 3 and 4 in Supplemental Material)

The non-carcinogenic health risk analyses for amaranth and fluted pumpkin samples are presented in Figure 5. In amaranth, hazard quotient values of 2.51, 12.69 and 7.70 were obtained for γ-HCH, *p, p′* DDT and methoxychlor, respectively, and γ-HCH (2.00), *p, p′* DDT (8.20) and methoxychlor (10.40) detected in fluted pumpkin had a hazard quotient (HQ) > 1, indicating a non-carcinogenic (systemic) health risk to children who consume these vegetables. For adults, the hazard quotient for methoxychlor (1.37) and DDT (2.25) were detected in amaranths while the HQs obtained for methoxychlor and DDT in fluted pumpkin were 1.86 and 1.40 respectively, which indicated a non-carcinogenic (systemic) health risk to adult consumers of these vegetables.

**Figure 6:**
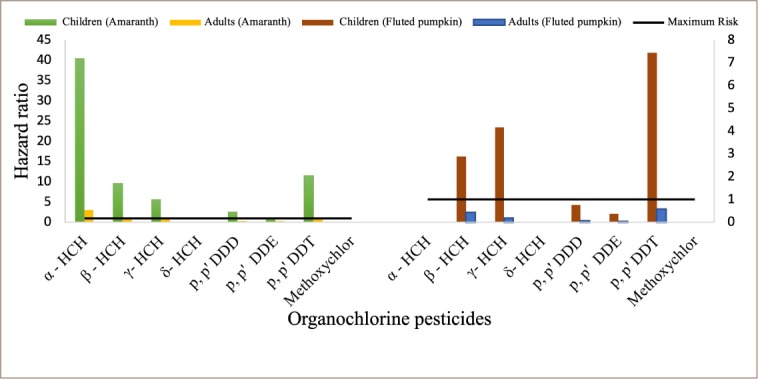
Potential carcinogenic health risk estimation of organochlorine pesticide residues in amaranth and fluted pumpkin from selected farms and markets in southwestern Nigeria (representing data presented in Tables 5 and 6 in Supplemental Material)

The carcinogenic health risk analysis for DDTs and HCHs detected in amaranth and fluted pumpkin is shown in Figure 6. For adult consumers, only α-HCH (3.066) detected in amaranth had hazard risk values > 1, indicating a carcinogenic risk to consumers with about 3 in 1 million adults at risk of developing cancer due to this exposure. For children, health ratio values were > 1 in α-HCH (40.426), *p, p′* DDT (11.454), β-HCH (9.625), γ-HCH (5.561) and *p, p′* DDD (2.567) detected in amaranth. All DDTs and HCHs detected in fluted pumpkin for adults had a hazard index < 1, indicating no carcinogenic risk to consumers or a risk below the maximum level of 1 in 1 million. Hazard ratio values of 7.454, 2.888 and 4.171 were obtained for *p, p′* DDT, β-HCH, and γ-HCH, respectively, indicating a carcinogenic health risk to children consuming fluted pumpkin.

## Discussion

The levels of organochlorine pesticide residues in amaranth and fluted pumpkin sampled from markets and farms followed similar trends of ∑HCH < ∑DDT, suggesting that similar pesticides were used on both vegetables for protection against insect pests. The δ-HCH was the most prominent HCH in amaranth samples, while γ-HCH and δ-HCH were the most predominant in fluted pumpkin. The high levels of δ-HCH from the two vegetables suggest the use of technical HCH, a commercial mixture of both γ and other isomers in which δ-HCH probably had the highest concentrations or as a result of degradation of γ-HCH and other isomers, while the predominance of γ-HCH in fluted pumpkin could be as a result of recent usage as it had not degraded to its metabolites of α-HCH and β-HCH.^16^

The ratio of α-HCH to γ-HCH can be used to monitor sources of HCHs. A ratio between 4–7 indicates technical HCH and a value of nearly zero indicates technical lindane.^11^,^31^ The ratio of α-HCH to γ-HCH detected in amaranth and fluted pumpkin in this study was lower than 4, suggesting that technical lindane was recently used or still being used for the control of insect pests in vegetables.

γ-HCH (lindane) commercially available as Gammalin 20 EC^®^, Gammalin super 20 EC^®^ and Capsitox 20 EC^®^ is already banned in Nigeria and worldwide. These pesticides and other organochlorine pesticides have been reported in vegetables in Nigeria and other countries of the world as shown in Table 2. Adeyeye and Osinbanjo and Oyeyiola *et al*. previously reported lower concentrations of DDTs and HCH isomers in vegetables from some locations in southwestern Nigeria and the levels reported by Ibrahim *et al*. were found to be comparable with the results obtained in the present study.^13^,^15^,^18^ However, in other countries like Ghana, China and Pakistan, the reported residue levels in vegetables were lower than those detected in the present study.^11^,^32^–^34^ In other food samples from Nigeria, pesticide residues detected in kolanut by Sosan and Oyekunle are comparable with the current study.^19^ Sosan *et al.* reported that the level of γ-HCH pesticide residues in cowpea grains in Ile-Ife market was 0.085 mg kg^−1^ and this value is within the range of γ-HCH detected in amaranth and fluted pumpkin from farms and markets in the study area, indicating that the pesticides were still being used as a general purpose pesticide for insect pest control.^16^ Ibigbami *et al*. previously reported the presence of 15 OCP residues, including α-HCH, β- HCH, lindane, *p, p′*-DDD and *p, p′*-DDT in water from the Ogbese river in Ekiti State.^35^ Ogbese is one of the most important flowing rivers in the study area. There is a high likelihood that farmers in this area use water from the river during dry season vegetable production, thereby contributing to the OCP residues detected in vegetable samples.

**Table 2:** Organochlorine Pesticides (DDT and HCHs) in Foods in Nigeria and Other Countries

**Country**	**Year**	**Foodstuff/commodity**	**Organochlorine pesticide residues (means in mg kg^−1^)**								**Reference**

			**α-HCH**	**β-HCH**	**γ-HCH**	**δ-HCH**	**DDD**	**DDE**	**DDT**	**Methoxychlor**	
**Nigeria** (southwestern Nigeria)	1999	Amaranth	-	-	0.0044	-	-	0.0011	0.0226		Adeyeye and Osinbanjo^13^
**Nigeria** (markets in Ile-Ife)	2015	Cowpea grain	0.019	0.047	0.085	0.062	0.159	0.073	0.053	0.042	Sosan *et al*,^16^
**Nigeria** (markets in Osun State)	2017	Kolanut	0.208	0.103	0.044	0.032	0.050	0.480	0.108	0.138	Sosan and Oyekunle^19^
**Nigeria** (Ile-Ife)	2017	Cocoa beans	0.41	0.86	0.47	0.63	7.71	0.83	57.76	35.04	Oyekunle *et al.*^11^
**Nigeria** (Nasarawa State)	2018	Fluted pumpkin	-	-	0.649	0.359	-	-	0.751	-	Ibrahim *et al.*^15^
**Nigeria** (markets in Lagos)	2018	Vegetables	0.00067	0.00162	0.00021	0.00003	0.00029	0.0885	0.00032	.	Oyeyiola *et al.*^18^
**Ghana**	2011	Vegetables	-	-	0.055	-	-	0.039	0.023	0.017	Bempah *et al.*^32^
**Ghana**	2018	Cabbage (Anglican Hostel)	-	0.0148	-	0.0079	-	-	0.0103	0.0090	Bolor *et al.*^33^
**China** (Deyang)	2009	Amaranth	0.0007	0.00010	0.00005	<0.00001	0.00022	0.00006	<0.00001	-	Owago *et al.*^11^
**Pakistan**	2018	Pumpkin	0.00020	0.00038	0.00062	-	0.00045	0.00039	-	-	Aamir *et al.*^34^

The prominence of methoxychlor and DDT in amaranth in the present study could be from the soil on which the vegetables were planted. For example, the presence of organochlorine pesticides was reported in soils from Oke-Osun farm settlement, which lies within one of the sample locations in Osun State.^20^ The presence of these two compounds in the vegetables could also be the result of non-target contamination from long range transportation of the OCPs applied to crops elsewhere.

DDT is biodegradable into DDD through reductive de-chlorination under anaerobic conditions and to DDE under aerobic conditions through de-hydrochlorination (an oxidative process).^36^ Ahmed *et al*. reported that the ratio of (DDE+DDD)/DDT >1 shows no recent inputs of DDT or degradation from previous residues.^37^ In the present study, amaranth and fluted pumpkin samples had ratios of (DDD+DDE)/DDTs of less than 1, meaning that DDT was recently used across the study locations as samples have not weathered or degraded. Usually, vegetables bought at farms are subjected to some degree of rinsing before being brought to market. In the process of rinsing, it is possible that some adsorbed DDT, HCHs and particles are washed off, especially if they have not yet absorbed into the vegetable (shoot system). This could be the reason for much lower pesticide residue levels found in amaranth from markets. Degradation of DDT to DDE is reported to be faster at higher temperatures, while conversion and isomerization of DDT could be caused by solar radiation from the sun.^38^ This might also play a part as most of harvested vegetables from farms are transported to the open markets where they are usually left under the heat of the sun as observed by Bempah and Donkor in a similar study in Ghana.^39^ This could be one of the reasons for lower DDT levels in amaranth samples from markets compared to farms. Another possible explanation could involve differences in the concentrations and pesticide active ingredients used at the various farms where the vegetables were grown.

All the HCHs and DDTs detected in the vegetables were above their respective maximum residue limits. The results of the present study corroborated those of earlier studies. For example, Akan *et al*. in northern Nigeria reported that concentrations of pesticides detected in five freshly harvested vegetables were observed to be significantly higher than MRLs set for vegetables; Sosan and Oyekunle reported organochlorine pesticide residues concentration detected in kolanut samples from selected markets in Osun State, southwestern Nigeria to be 44%–92% above EU-MRLs for DDTs and 76%–100% above EU-MRLs for HCH isomers.^14^,^19^ In another study, Sosan *et al.* reported that levels of lindane (γ-HCH) detected in all cowpea samples and 90% of dried yam chips samples were above the recommended EU-MRL of 0.01 mg kg^−1^, which was higher than the present study.^16^ However, in another study, the concentrations of all the pesticides detected in the fruit and vegetable samples from markets within Kaduna Metropolis were established to be considerable lower than the EU-MRLs.^40^ The presence of residues above MRLs indicated that high concentrations of the pesticides were still being used and detected in our environment with the possibility of causing systemic toxicity for the regular consumers of the two vegetables.

Methoxychlor, γ-HCH and DDT posed a non-carcinogenic threat or risk to children consumers of the selected vegetables, while only methoxychlor and DDT posed a non-carcinogenic threat or risk to adult consumers of contaminated amaranth and fluted pumpkin. In comparison with earlier studies *(Table 3)*, the hazard risk values in the present study were higher than those of vegetables (cabbage, cameroon pepper, green pepper, chili pepper and lettuce) collected from selected markets in Lagos, Nigeria which posed no risk to adults or children.^18^ The hazard index reported for adult consumers of vegetables in Ghana by Bempah *et al.* was lower compared to the current study.^32^ In addition, the estimated HQ in this study was higher than those for both children and adult consumers of cabbage vegetables from the Anglican Hostel location in Ghana reported by Bolor *et al*.^33^ The results obtained from the present study were lower compared with the analysis of health risk estimates of pesticides in kolanut obtained from markets in Osun State which had HQ values of > 1 for γ-HCH.^19^ For carcinogenic risk, the hazard ratio for child consumers of the two vegetables in the present study are comparable with the results of studies by Aamir *et al*. and Wang *et al*. in Pakistan and Cambodia, respectively, which reported hazard ratios greater than 1 for adult consumers of vegetables.^29^,^34^

**Table 3:** Non-Cancer and Cancer Risk of DDTs and HCHs Detected in Various Foods in Nigeria and Vegetables Across Countries

**Country**	**Year**	**Foodstuff/Commodity**		**Non-cancer and cancer risk for adults and children**								**Reference**

				**α-HCH**	**β-HCH**	**γ-HCH**	**δ-HCH**	**DDD**	**DDE**	**DDT**	**Methoxychlor**	
**Nigeria** (markets in Osun State)	2017	Kolanut	**Non-carc.** (adult)	0.338	-	1.907	0.138	0.003	0.312	0.070	0.358	Sosan and Oyekunle^19^
			**Non-carc.** (adult)	< 1	< 1	< 1	< 1	< 1	< 1	< 1	-	
**Nigeria** (markets in Lagos)	2018	Vegetables	**Non-carc.** (children)	< 1	< 1	< 1	< 1	< 1	< 1	< 1	-	Oyeyiola *et al.*^18^
**Ghana**	2011	Vegetables	**Non-carc.** (adult)	-	-	0.160		-	0.054	0.016	0.130	Bempah *et al.*^32^
**Ghana**	2018	Cabbage (Anglican Hostel)	**Non-carc.** (adult)	-	0.0112	-	0.0060	-	-	0.0012	0.0041	Bolor *et al.*^33^
			**Non-carc.** (children)	-	0.0674	-	0.0361	-	-	0.0071	0.0247	
**Cambodia**	2011	Vegetable	**Carc.** (adult)	186	2.82	2.44	-		4.62	2.52	-	Wang *et al.*^29^
**Pakistan**	2018	Vegetables	**Carc.** (adult)	11.9	4.01	1.71	-	-	0.80	0.74	-	Aamir *et al*^34^

Abbreviations: non-carc., non-carcinogenic risk; carc., carcinogenic risk.

Consumption of the γ-HCH-contaminated amaranth and fluted pumpkin could cause liver and kidney toxicity for child consumers of these vegetables. Consumption of both vegetables contaminated with DDT and methoxychlor by children and adult consumers could cause liver lesions as well as disrupt reproductive and developmental functions.^27^,^41^,^42^ The International Agency for Research on Cancer classified both HCH isomers and DDTs as possibly carcinogenic to humans.^43^ Both DDTs and HCHs detected in this study could pose carcinogenic risk (notably liver tumor, hepatic nodules, hepatocellular carcinomas and neoplastic nodules) to child consumers of the vegetables, while α-HCH could pose carcinogenic risk (hepatic nodules and hepatocellular carcinomas) to adult consumers of amaranths.^27^ Hence, there is possibility of child consumers of both vegetables and adult consumers of amaranth contaminated with α-HCH developing liver cancer over their lifetime. Continuous exposure via vegetable consumption could lead to higher potential for both non-carcinogenic and carcinogenic risks.

## Conclusions

The present study revealed a high occurrence of DDTs and HCHs in amaranth and fluted pumpkin from the selected locations in southwestern Nigeria with contamination levels greater than their respective MRLs. The hazard ratio values for adults revealed that exposure through consumption of amaranth contaminated with α-HCH poses carcinogenic health risks. The estimate suggested a potential non-carcinogenic and carcinogenic health risk for children and adult consumers of the two vegetables. If pesticides used in production of vegetables in Nigeria are not regulated, there is a possibility of an upsurge in potential risks with resultant health hazards associated with their usage. Strict enforcement of regulations on pesticide usage and regular monitoring of pesticide residues in food products in Nigeria is needed to minimize health risks.
