# Supplementary material for: Occurrence and Human Health Risk of Dichlorodiphenyltrichloroethane (DDT) and Hexachlorocyclohexane (HCH) Pesticide Residues in Commonly Consumed Vegetables in Southwestern Nigeria
Source: J Health Pollut. 2019 Aug 6;9(23):190909. doi: 10.5696/2156-9614-9.23.190909 (PMC6711335; doi:10.5696/2156-9614-9.23.190909)
Supplement: Supplementary file 1 [file Adeleye_Supplemental_Material_FINAL.docx]

**Supplemental Material**

**Table of contents**

Table 1: Mean Concentrations of DDT and HCH Pesticide Residues on *Amaranthus* spp. and Fluted Pumpkin from Selected Markets and Farms in Southwestern Nigeria

Table 2: Mean Concentrations and MRLs of OCPs Residues in Amaranths and Fluted Pumpkin from Markets and Farms from Selected Locations in Southwestern Nigeria

Table 3: Potential Non-Carcinogenic Health Risk Estimation of Organochlorine Residues in Amaranths from Selected Farms and Markets in Southwestern Nigeria

Table 4: Potential Non-Carcinogenic Health Risk Estimation of Organochlorine Pesticide Residues in Fluted Pumpkin from Selected Farms and Markets in Southwestern Nigeria

Table 5: Potential Carcinogenic Health Risk Estimation of Organochlorine Pesticide Residues in *Amaranthus* spp. from Selected Farms and Markets in Southwestern Nigeria

Table 6: Potential Carcinogenic Health Risk Estimation of Organochlorine Pesticide Residues in Fluted Pumpkin from Selected Farms and Markets in Southwestern Nigeria

Table 7: Concentration/Levels of Organochlorine Pesticide Residues in *Amaranthus* spp. from Selected Farms in Southwestern Nigeria (Raw Data)

Table 8: Concentration/Levels of Organochlorine Pesticide Residues in *Amaranthus* spp. from Selected Markets in Southwestern Nigeria (Raw Data)

Table 9: Concentration/Levels of Organochlorine Pesticide Residues in Fluted Pumpkin from Selected Farms in Southwestern Nigeria (Raw Data)

Table 10: Concentration/Levels of Organochlorine Pesticide Residues in Fluted Pumpkin from Selected Markets in Southwestern Nigeria (Raw Data)

**Amaranthus (n=16)     Fluted pumpkin (n=16)**

**Farms     Markets Farms   Markets**

**Pesticides** Mean ± SE % Occurrence Mean ± SE % Occurrence Mean ± SE % Occurrence Mean ± SE %Occurrence

(mg kg^-1^) (mg kg^-1^) (mg kg^-1^) (mg kg^-1^)

α-HCH 0.286 ± 0.286 12.5 ND ND ND ND ND ND

β-HCH 0.246 ± 0.099 87.5 ND ND 0.010 ± 0.010 12.5 0.072 ± 0.072 12.5

γ-HCH 0.102 ± 0.030 87.5 0.077 ± 0.024 75.0 0.086 ± 0.016 87.5 0.064 ± 0.021 62.5

δ-HCH 0.986 ± 0.448 87.5 0.168 ± 0.068 62.5 0.542 ± 0.080 100.0 0.467 ± 0.147 100.0

**Ʃ HCH 1.348 ± 0.561**   **0.217 ± 0.086**   **0.638 ± 0.092**   **0.603 ± 0.163**

p, p' DDD 0.258 ± 0.195 62.5 0.220 ± 0.210 25.0 0.114 ± 0.114 12.5 0.039 ± 0.027 25.0

p, p' DDE 0.094 ± 0.025 75.0 0.034 ± 0.023 25.0 0.039 ± 0.022 37.5 0.011 ± 0.011 12.5

p, p' DDT 1.251 ± 0.737 62.5 0.263 ± 0.263 12.5 0.254 ± 0.174 37.5 0.718 ± 0.203 75.0

Methoxychlor 8.536 ± 4.428 100 0.643 ± 0.365 37.5 6.458 ± 1.821 87.5 5.988 ±2.533 75.0

**ƩDDT** **10.139 ± 4.576** **1.159 ± 0.656** **6.865 ± 1.742** **6.756 ± 2.543**

**OCI burden 11.488 ± 4.794**  **1.376 ± 0.672**  **7.502 ± 1.736**  **7.359 ± 2.638**

**p-value (Mann-Whitney)**   **0.** **0458***   **0**. **9588ns**

**Table 1: Mean Concentrations of DDT And HCH Pesticide Residues on *Amaranthus* spp. and Fluted Pumpkin from Selected Markets and Farms in Southwestern Nigeria**

Abbreviations: ND, not detected; SE, standard error.
* = significant at α = 0.05; ns = not significant at α = 0.05
**(Represent data presented in Figures 2 and 3)**

**Amaranthus (n=16) Fluted pumpkin (n=16)**

**Pesticides** UK/EC MRL Mean ± SE Range % Above MRLs % Group Mean ± SE Range % Above MRLs % Group

(mg kg^-1^) (mg kg^-1^) (mg kg^-1^) (mg kg^-1^) (mg kg^-1^)

α-HCH 0.01 0.143 ±0.143 ND - 2.287 6.3 ND ND ND

β-HCH 0.01 0.123 ± 0.030 ND - 0.911 43.8 0.041 ± 0.031 ND - 0.579 12.5

γ-HCH 0.01 0.09 ± 0.031 ND - 0.210 81.3 0.075 ± 0.002 ND - 0.158 75.0

δ-HCH 0.01 0.577±0.390 ND - 3.868 75.0 0.504 ± 0.056 0.048 - 1.223 100.0

**Ʃ HCH**  **0.933 ± 0.594**   **14.2**  **0.62 ± 0.090**   **8.3**

**α-HCH / γ-HCH**  **1.6**  **0**

p, p' DDD 0.05 0.239 ± 0.016 ND - 1.692 43.8 0.077 ± 0.038 ND - 0.915 18.8

p, p' DDE 0.05 0.063 ± 0.001 ND - 0.218 50.0 0.025 ± 0.005 ND - 0.173 25.0

p, p' DDT 0.05 0.757 ± 0.457 ND - 6.201 37.5 0.486 ± 0.123 ND - 1.652 56.3

Methoxychlor 0.01 4.59 ± 2.774 ND - 38.349 68.8 6.223 ± 2.489 ND - 21.198 81.3

**ƩDDT 5.649 ± 3.249** **85.8 6.81 ± 1.489 91.7**

**(DDE+DDD)/DDT 0.4 0.2**

**Total OCI burden 6.582**   **7.43**

**(DDE+DDD)/DDT 0.4**   **0.2**

**Table 2: Mean Concentrations and MRLs of OCPs Residues in Amaranths and Fluted Pumpkin from Markets and Farms from Selected Locations in Southwestern Nigeria**

Abbreviations: ND, not detected; SE, standard error.
**(Represent data presented in Figure 4)**

**Table 3: Potential Non-Carcinogenic Health Risk Estimation of Organochlorine Residues in Amaranths from Selected Farms and Markets in Southwestern Nigeria**

| **Pesticides** | **RfD** | **Children** | | | **Adult** | | | |
| --- | --- | --- | --- | --- | --- | --- | --- | --- |
|  | (mg kg^-1^d^-1^) | EADI (mg kg^-1^d^-1^) | Hazard quotient | Health risk | EADI (mg kg^-1^d^-1^) | | Hazard index | Health risk |
| α – HCH | - | 1.2 x 10^-3^ | - | - | 2.1 x 10^-4^ | - | |  |
| β-HCH | - | 1.0 x 10^-3^ | - | - | 1.8 x 10^-4^ | - | |  |
| γ- HCH | 0.0003^a^ | 7.6 x 10^-4^ | **2.51** | **Yes** | 1.3 x 10^-4^ | 0.45 | | No |
| δ-HCH | - | 4.8 x 10^-3^ | - | - | 8.6 x 10^-4^ | - | | - |
| p, p' DDD | - | 2.0 x10^-3^ | - | - | 3.6 x 10^-4^ | - | | - |
| p, p' DDE | - | 5.2 x10^-4^ | - | - | 19.3 x 10^-5^ | - | | - |
| p, p' DDT | 0.0005^b^ | 6.4 x10^-3^ | **12.69** | **Yes** | 1.1 x 10^-3^ | **2.25** | | **Yes** |
| Methoxychlor | 0.005^c^ | 3.9 x10^-2^ | **7.70** | **Yes** | 6.8 x 10^-3^ | **1.37** | | **Yes** |

Abbreviations: RfD, reference dose; EADI, estimated average daily intake.

**^a^** reference dose for liver and kidney toxicity.
**^b^** reference dose for liver lesions.

**^c^** reference dose for reproductive and developmental effects.**^27^**

**(Represent data presented in Figure 5)**

**Table 4: Potential Non-Carcinogenic Health Risk Estimation of Organochlorine Pesticide Residues in Fluted Pumpkin from Selected Farms and Markets in Southwestern Nigeria**

|  | **RfD** | **Children** |  |  | **Adult** |  |  |
| --- | --- | --- | --- | --- | --- | --- | --- |
| **Pesticides** | (mg kg-1d-1) | EADI (mg kg-1d-1) | Hazard quotient | Health risk | EADI (mg kg-1d-1) | Hazard index | Health risk |
| α – HCH | - | - | - | - | - | - | - |
| β-HCH | - | 3.0 x 10^-4^ | - | - | 1.0 x 10^-4^ | - | - |
| γ- HCH | 0.0003^a^ | 6.0 x 10^-4^ | **2.00** | **Yes** | 6.0 x 10^-5^ | 0.20 | No |
| δ-HCH | - | 4.2 x 10^-3^ | - | - | 8.0 x 10^-4^ | - | - |
| p, p' DDD | - | 6.0 x 10^-4^ | - | - | 1.0 x 10^-4^ | - | - |
| p, p' DDE | - | 2.0 x 10^-4^ | - | - | 4.0 x 10^-5^ | - | - |
| p, p' DDT | 0.0005^b^ | 4.1 x 10^-3^ | **8.20** | **Yes** | 7.0 x 10^-4^ | **1.40** | **Yes** |
| Methoxychlor | 0.005^c^ | 5.2 x 10^-2^ | **10.40** | **Yes** | 9.3 x 10^-3^ | **1.86** | **Yes** |

Abbreviations: RfD, reference dose; EADI, estimated average daily intake.

**^a^** reference dose for liver and kidney toxicity.

**^b^** reference dose for liver lesions.

**^c^** reference dose for reproductive and developmental effects.**^27^**

**(Represent data presented in Figure 5)**

**Table 5: Potential Carcinogenic Health Risk Estimation of Organochlorine Pesticide Residues in *Amaranthus* spp. from Selected Farms and Markets in Southwestern Nigeria**

|  | **Children** | | | **Adult** | | |
| --- | --- | --- | --- | --- | --- | --- |
| **Pesticides** | CBC | HR | Health risk | CBC | HR | Health risk |
| α – HCH | 2.97 x 10^-5^ | 40.426 | **Yes** | 6.52 x 10^-5^ | 3.066 | **Yes** |
| β – HCH | 1.04 x 10^-4^ | 9.625 | **Yes** | 2.28 x 10^-4^ | 0.876 | No |
| γ- HCH | 1.44 x 10^-4^ | 5.561 | **Yes** | 3.16 x 10^-4^ | 0.633 | No |
| δ- HCH | - | - | - | - | - | - |
| *p, p'* DDD | 7.79 x 10^-4^ | 2.567 | **Yes** | 1.71 x 10^-3^ | 0.234 | No |
| *p, p'* DDE | 5.50 x 10^-4^ | 0.909 | No | 1.21 x 10^-3^ | 0.331 | No |
| *p, p'* DDT | 5.50 x 10^-4^ | 11.454 | **Yes** | 1.21 x 10^-3^ | 0.910 | No |
| Methoxychlor | - | - | - | - | - | - |

Abbreviations: HR, hazard ratio; CBC, cancer benchmark concentration

**(Represent data presented in Figure 6)**

**Table 6: Potential Carcinogenic Health Risk Estimation of Organochlorine Pesticide Residues in Fluted Pumpkin from Selected Farms and Markets in Southwestern Nigeria**

|  | **Children** | | | **Adult** | | | | |  |
| --- | --- | --- | --- | --- | --- | --- | --- | --- | --- |
| **Pesticides** | CBC | HR | Health risk | CBC | HR | | | Health risk |  |
| α – HCH | 4.16 x 10^-5^ | - | - | 6.52 x 10^-5^ | - | | - | | |
| β – HCH | 1.04 x 10^-4^ | 2.888 | **Yes** | 2.28 x 10^-4^ | 0.438 | | No | | |
| γ- HCH | 1.44 x 10^-4^ | 4.171 | **Yes** | 3.16 x 10^-4^ | 0.190 | | No | | |
| δ- HCH | - | - | - | - | - | | - | | |
| *p, p'* DDD | 7.79 x 10^-4^ | 0.770 | No | 1.71 x 10^-3^ | 0.058 | | No | | |
| *p, p'* DDE | 5.50 x 10^-4^ | 0.364 | No | 1.21 x 10^-3^ | 0.033 | | No | | |
| *p, p'* DDT | 5.50 x 10^-4^ | 7.454 | **Yes** | 1.21 x 10^-3^ | 0.579 | | No | | |
| Methoxychlor | - | - | - | - | - | - | | |  |

Abbreviations: HR, hazard ratio; CBC, cancer benchmark concentration

**(Represent data presented in Figure 6)**

**Raw Data**

**Table 7:** **Concentration/Levels of Organochlorine Pesticide Residues in *Amaranthus* spp. from Selected Farms in Southwestern Nigeria**

| **Pesticides** | **AFE1** | **AFE2** | **AFE3** | **AFE4** | **AFO1** | **AFO2** | **AFO3** | **AFO4** |
| --- | --- | --- | --- | --- | --- | --- | --- | --- |
| α-HCH | ND | ND | ND | ND | ND | ND | 2.2873 | ND |
| β-HCH | 0.1359 | 0.1792 | 0.9110 | ND | 0.1052 | 0.1768 | 0.1743 | 0.2840 |
| γ- HCH | ND | 0.2074 | 0.0542 | 0.0588 | 0.0488 | 0.2103 | 0.1829 | 0.0502 |
| δ-HCH | 0.2001 | 0.8305 | ND | 0.3074 | 0.6169 | 3.8683 | 0.4347 | 1.6271 |
| p, p' DDD | ND | ND | 0.0988 | 1.6157 | ND | 0.1066 | 0.1456 | 0.0941 |
| p, p' DDE | 0.0769 | 0.0977 | ND | 0.2184 | ND | 0.1263 | 0.1210 | 0.1096 |
| p, p' DDT | ND | 0.3470 | 6.2008 | 1.0672 | ND | ND | 1.6161 | 0.7800 |
| Methoxychlor | 1.3720 | 2.3608 | 7.7152 | 0.7431 | 10.0033 | 1.3544 | 38.3494 | 6.3937 |

Abbreviations: AFE, amaranths samples from farms in Ekiti State; AFO, amaranths samples from farms in Osun State; ND, not detected and below detection limits of 0.0069 - 0.0102 mg kg^-1^.

**Table 8:** **Concentration/Levels of Organochlorine Pesticide Residues in *Amaranthus* spp. from Selected Markets in Southwestern Nigeria**

| **Pesticides** | **AMO1** | **AMO2** | **AMO3** | **AMO4** | **AME1** | **AME2** | **AME3** | **AME4** |
| --- | --- | --- | --- | --- | --- | --- | --- | --- |
| α – HCH | ND | ND | ND | ND | ND | ND | ND | ND |
| β-HCH | ND | ND | ND | ND | ND | ND | ND | ND |
| γ- HCH | 0.0626 | 0.1921 | 0.0570 | 0.1568 | ND | 0.0600 | 0.0901 | ND |
| δ-HCH | 0.5766 | 0.2658 | 0.1533 | 0.1937 | ND | ND | 0.1559 | ND |
| p, p' DDD | ND | 1.6919 | ND | ND | ND | ND | 0.0651 | ND |
| p, p' DDE | 0.1607 | ND | ND | ND | ND | ND | 0.1097 | ND |
| p, p' DDT | ND | ND | ND | ND | ND | ND | 2.1009 | ND |
| Methoxychlor | ND | 2.8127 | ND | ND | 0.9045 | ND | 1.4260 | ND |

Abbreviations: AME, amaranth samples from markets in Ekiti State; AMO, amaranth samples from markets in Osun State; ND, not detected and below detection limits of 0.0069 - 0.0102 mg kg^-1^.

**Table 9: Concentration/Levels of Organochlorine Pesticide Residues in Fluted Pumpkin from Selected Farms in Southwestern Nigeria**

| **Pesticides** | **UFE1** | **UFE2** | **UFE3** | **UFE4** | **UFO1** | **UFO2** | **UFO3** | **UFO4** |
| --- | --- | --- | --- | --- | --- | --- | --- | --- |
| α – HCH | ND | ND | ND | ND | ND | ND | ND | ND |
| β-HCH | ND | ND | ND | ND | ND | ND | ND | 0.081 |
| γ- HCH | 0.093308 | 0.15831 | 0 | 0.088611 | 0.063 | 0.091 | 0.085 | 0.107 |
| δ – HCH | 0.366289 | 0.4588 | 0.3625 | 0.795264 | 0.654 | 0.407 | 0.351 | 0.940 |
| *p, p'* DDD | 0.914697 | ND | ND | ND | ND | ND | ND | ND |
| *p, p'* DDE | ND | ND | ND | 0.067589 | ND | 0.069 | 0.000 | 0.173 |
| *p, p'* DDT | ND | 1.42033 | ND | ND | ND | ND | 0.251 | 0.358 |
| Methoxychlor | 6.51737 | 3.42189 | 10.12064 | 10.34931 | 14.824 | 5.981 | ND | 0.450 |

Abbreviations: PFE, pumpkin samples from farms in Ekiti State; PFO, fluted pumpkin samples from farms in Osun State; ND, not detected and below detection limits of 0.0069 to 0.0102 mg kg^-1^.

**Table 10: Concentration/Levels of Organochlorine Pesticide Residues in Fluted Pumpkin from Selected Markets in Southwestern Nigeria**

| **Pesticides** | **UMO1** | **UMO2** | **UMO3** | **UMO4** | **UME1** | **UME2** | **UME3** | **UME4** |
| --- | --- | --- | --- | --- | --- | --- | --- | --- |
| α – HCH | ND | ND | ND | ND | ND | ND | ND | ND |
| β-HCH | ND | ND | ND | ND | ND | ND | 0.579 | ND |
| γ- HCH | 0.081 | ND | 0.133 | 0.058 | 0.113 | ND | ND | 0.128 |
| δ – HCH | 0.339 | 0.786 | 0.060 | 0.048 | 1.222 | 0.123 | 0.440 | 0.714 |
| *p, p'* DDD | 0.115 | 0.196 | ND | ND | ND | ND | ND | ND |
| *p, p'* DDE | ND | ND | ND | ND | 0.088 | ND | ND | ND |
| *p, p'* DDT | 0.629 | 1.314 | 1.652 | 0.664 | 0.877 | ND | ND | 0.609 |
| Methoxychlor | 2.499 | 5.048 | 1.074 | ND | 21.198 | 9.425 | ND | 8.661 |

Abbreviations: UME, fluted pumpkin samples from markets in Ekiti State; UMO, fluted pumpkin samples from markets in Osun State; ND, not detected and below detection limits of 0.0069 to 0.0102 mg kg^-1^.
